# Influence of Hole Localization on Local and Global Dynamic Response of Thin-Walled Laminated Cantilever Beam

**DOI:** 10.3390/ma14237409

**Published:** 2021-12-03

**Authors:** Marcin Bochenski, Jaroslaw Gawryluk, Andrzej Teter

**Affiliations:** Department of Applied Mechanics, Faculty of Mechanical Engineering, Lublin University of Technology, Nadbystrzycka Str. 36, 20-618 Lublin, Poland; m.bochenski@pollub.pl (M.B.); a.teter@pollub.pl (A.T.)

**Keywords:** thin-walled structure, laminate, CUS beam, experimental modal analysis, eigenvalues, hole-like defect, closed cross-section

## Abstract

In this study, we discuss the effects of the diameter and position of a hole on the dynamic response of a thin-walled cantilever beam made of carbon-epoxy laminate. Eigen-frequencies and corresponding global and local eigen-modes were considered, where deformations of the beam wall were dominant, without significant deformation of the beam axis. The study was focused on the circumferentially uniform stiffness (CUS) beam configuration. The laminate layers were arranged as [90/15(3)/90/15(3)/90]_T_. The finite element method was employed for numerical tests, using the Abaqus software package. Moreover, a few numerical results of the structure’s behaviour, with and without a hole, were verified experimentally. The experimental eigen-frequencies and the corresponding modes were obtained using an experimental modal analysis, comprising the LMS system with modal hammer. We found that the size and location of the hole affected the eigen-frequencies and corresponding modes. Furthermore, even a small hole in a beam could significantly change the shape of its local modes. The numerical and experimental results were observed to have high qualitative compliance.

## 1. Introduction

Thin-walled laminate structures with closed cross-sections are commonly used elements in aerospace, mechanical and civil engineering, for example, in aircraft wings, helicopter and wind turbine blades, etc. They operate in complex environmental conditions and are exposed to various dynamic excitations. Laminate structures have a high strength-to-weight ratio, but they are sensitive to different types of damage. Three types of defects are commonly found in these structures—classical delamination, delamination parallel to the fibre orientation, and hole in the beam surface. A hole could appear in the structure because of high energy impact [1] and reduce its stiffness and inertia. Changing these parameters influences the structure’s dynamic response. However, this type of damage is insufficiently described in the literature. The size and location of damage can be determined by applying appropriate algorithms. Damage detection methods for laminated structures are mostly based on:

(a) changing values of the eigen-frequencies,
(b) changing the shape of eigen-modes,
(c) changing energy of strain [2,3],
(d) ultrasonic testing [4],
(e) fibre Bragg grating [5], and
(f) change in elastic wave propagation [6].

In paper [7], the authors compared the natural frequencies of healthy and delaminated laminated plates using the free vibration test. Using the finite element method (FEM) and experimental studies, they found that the influence of defects is more evident for higher modes than for lower. In paper [8], a similar technique was used to detect the size and location of several simultaneous damages. The dynamics of beams with multiple delamination were tested in [9]. Experimental and analytical approaches were employed, and nonlinear behaviour was confirmed. In paper [10], the nonlinear vibrations of composite beams subjected to harmonic excitation were determined, which were further used to study the sensitivity of selected vibration response parameters to damage. Therein, the method based on Poincare maps was used. A different type of damage was considered in [11], namely delamination, debonding fatigue and drilled hole. The authors argued that, based on the change in frequency, it was difficult to determine the extent of the damage, if its type and location were unknown. Sometimes, a small hole in the beam causes a spectrum shift similar to delamination, of nearly ten times the area. In paper [12], the degradation of the stiffness of a laminated plate under the influence of a macro-level crack was considered. The authors concluded that the modal strain energy and curvature mode shape had higher sensitivity to damage than natural frequencies and displacement mode shapes. The hole-type defect was analysed in [5]. Using the fibre Bragg grating, the relationship between strain and damage in laminated plates was found. The results obtained by the proposed method were in good agreement with those of an ultrasonic fault detector. Detecting a hole-type damage is very important, because fatigue failures could start at the edges of such a hole [6]. The detection of through-holes in a laminated plate using Lamb wave was presented in paper [13]. The method used could detect damages as small as 1 mm in diameter if appropriate features were chosen, but deviation from the model of normality could lead to false damage indication. When techniques based on the analysis of the dynamic response of the system are used, the excitation amplitude and the level of generated vibrations of the structure are crucial. Higher amplitudes contribute to intensification of nonlinear phenomena and facilitate damage detection [14].

In this study, the impact of a hole-type damage on the dynamic response of a thin-walled laminated beam was investigated using small inputs generated by a modal hammer. This method is often used to test composite structures [15,16]. Similar conditions are met when an eigenvalue problem is solved for a linear model using FEM, and the eigen-frequencies and the global and local eigen-modes are considered. Understanding the behaviour of this structure will allow for detecting defects of this type in the future, without interrupting the operation of the system. The object of the study is presented in Section 2, and Section 3 includes a description of the creation of a numerical FEM model. Furthermore, the experimental tests of the analysed specific cases of the cantilever box beam are presented in Section 4. Section 5 contains all the test results, which are summarized in Section 6.

## 2. Object of the Study

**Figure 1:**
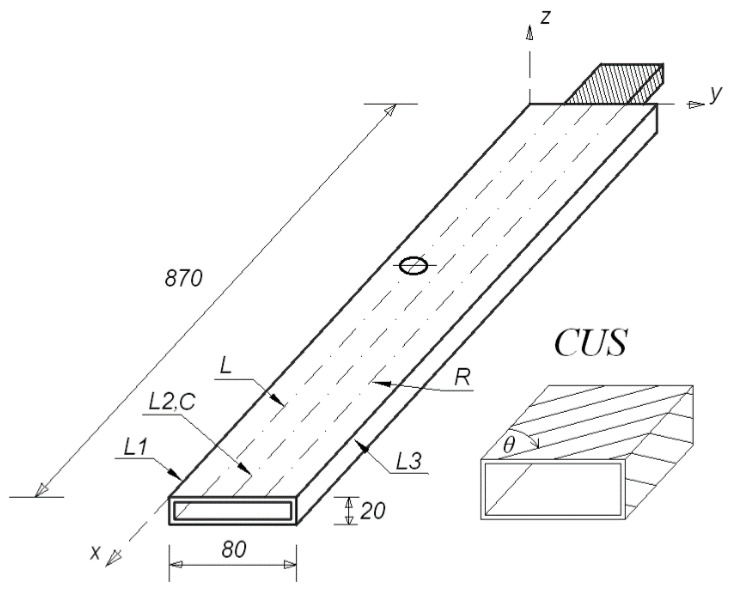
Dimensions of the laminated box-beam.

A laminated box-beam made of unidirectional carbon-epoxy prepreg with circumferentially uniform stiffness (CUS) beam configuration was considered. Herein, the structure’s vibrations were decoupled into two independent couplings—bending in stiff and flexible direction, and torsion with extension [17]. The laminate layer were arranged as [90/15(3)/90/15(3)/90]_T_. The 15° angle was optimized to obtain the strongest bending coupling coefficient [18]. The laminate material constants given by the manufacturer of the samples were: longitudinal Young’s modulus: 143.2 GPa, transversal Young’s modulus: 3.1 GPa, Poisson’s ratio: 0.35, shear modulus: 3.38 GPa, mass density: 1442 kg/m^3^. Figure 1 shows the dimensions of the analysed structure. The length of the beam was 870 mm. The dimensions of its rectangular cross section were 80 mm × 20 mm. The thickness of each wall of the beam was equal to 0.51 mm, but measurements at the edges of the fabricated beam showed small differences in wall thickness. The fixed grip was made of 6082 aluminium alloy.

The numerical–experimental studies were carried out for a structure without a hole (Case1), and with a hole (Case2). The structure in Case2 had a single hole made in the upper wall of the beam, and various diameters and locations of the hole were considered. Holes with a radius of 4, 8, 12 and 16 mm, located on three lines: left, centre and right (denoted in Figure 1 as L, C, R, respectively) at distances of 150, 350, 550 and 750 mm from the fixed grip, respectively, were analysed numerically. The left and right lines were located 20 mm from the centre line. Forty-eight different configurations of the location and the size of the hole were examined using numerical tests. However, a few cases were selected for experimental verification.

## 3. Finite Element Method (FEM)

**Figure 2:**
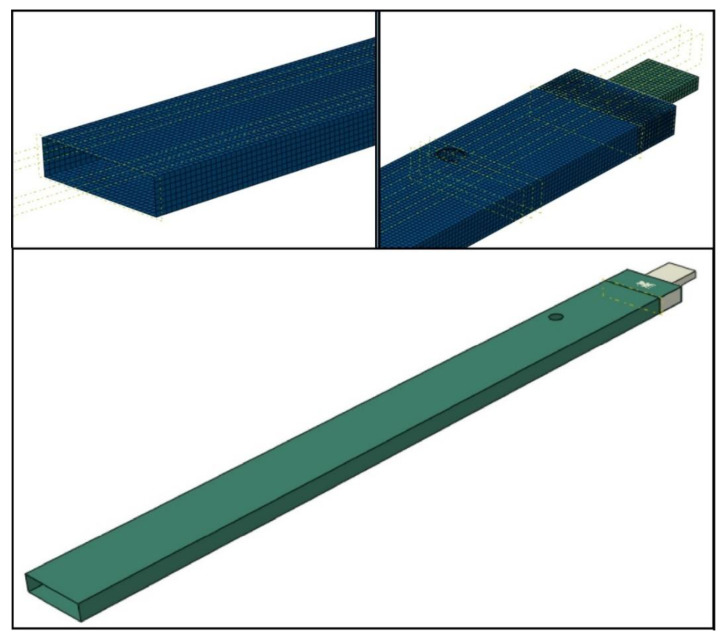
FE model of the beam with a hole.

The numerical FE-model of the beam (with and without the hole) comprised shell finite elements. These are the elements of the S8R type having reduced integration [19]. They are 8-node elements having a second-order shape function with reduced integration (having six degrees of freedom in each node). The arrangement of the laminate layers was prepared using the layup-ply technique. The beam was fixed on one end of the grip, which was modelled using the C3D20R-type elements. These are 20-node second-order elements (with square shape function) with three translational degrees of freedom at each node. Figure 2 shows the developed numerical model of the system. The mechanical boundary conditions were realized by restraining all translational degrees of freedom of the nodes at the grip. The beam was attached to the grip using TIE interactions [20]. These interactions enabled the simulation of a permanent connection of both elements. The number of elements was assumed based on existing knowledge, such as [21,22]. The convergence of the model was investigated numerically, by selecting the size of the elements used. However, the mesh density was assumed such that the beam deformation was not limited. In this model, the mesh consisted of 22,276 elements (quadratic quadrilateral elements of type S8R, and quadratic hexahedral elements of type C3D20R). The Lanczos method was used for solving the eigenvalue problem, to determine the eigen-frequencies and corresponded mode [23]. It is a commonly used method that was successfully presented in [22]. Numerical studies were carried out in the Abaqus environment, where the eigenvalue problem was determined for all ana lysed variants.

## 4. Experimental Modal Analysis

**Figure 3:**
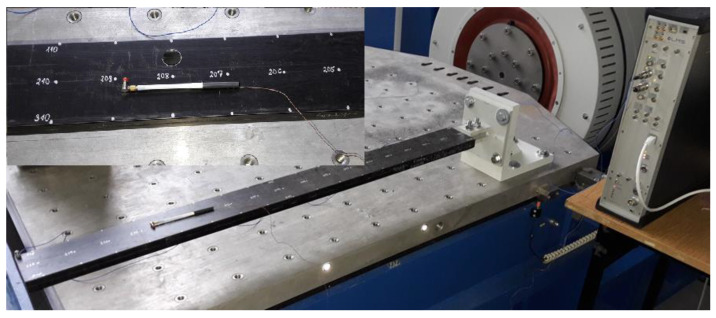
Experimental stand.

**Figure 4:**
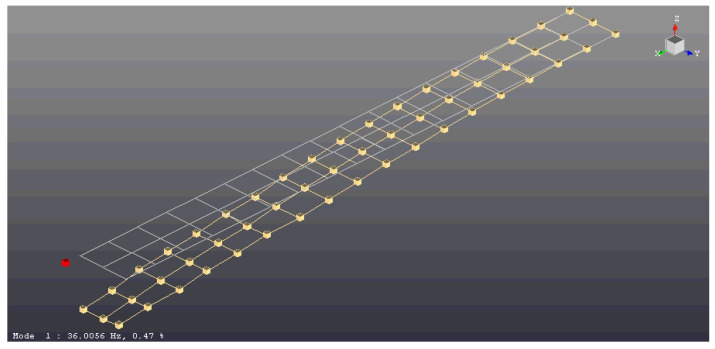
Distribution of measurement points on the beam—geometry generated in TestLab software.

For the experimental analysis, the discussed laminated beam was pasted on the grip and fixed to a heavy platform (Figure 3). A PCB 086E80 miniature impulse hammer, PCB 352B10 lightweight accelerometer, and LMS SCADAS modal analyser with TestLab software were used. Figure 4 shows the model of the beam geometry created in the TestLab software, which contained 54 measuring points (shown in beige). The location of the accelerometer was denoted using a red point. Measurement points were placed on lines denoted as L1, L2 and L3 (see Figure 1). The L2 line was on the beam axis, while the L1 and L3 lines were on its edges. Points on the centre L2 line were impacted only in the vertical *z*-direction; however, points on the L1 and L3 lines were impacted from both the *y-* and *z*-directions. Each hit was repeated five times, and the result was based on the average response. All incorrect measurements were rejected. Too weak or too strong of an impact was detected by the software, but double impact could be recognised by the user, from the frequency response function (FRF) graph. Additionally, observation of the coherence graph allowed us to determine whether the measurement was repeatable. First, eigen-frequencies and eigen-modes were determined for the beam without the hole. Then, at a distance of 350 mm from the grip on the L line, a hole with a radius of 8 mm was made and the procedure was repeated. This case was denoted as beam CUS 350L R8. Figure 3 also shows a part of the beam with the prepared hole.

## 5. Results and Discussion

**Figure 5:**
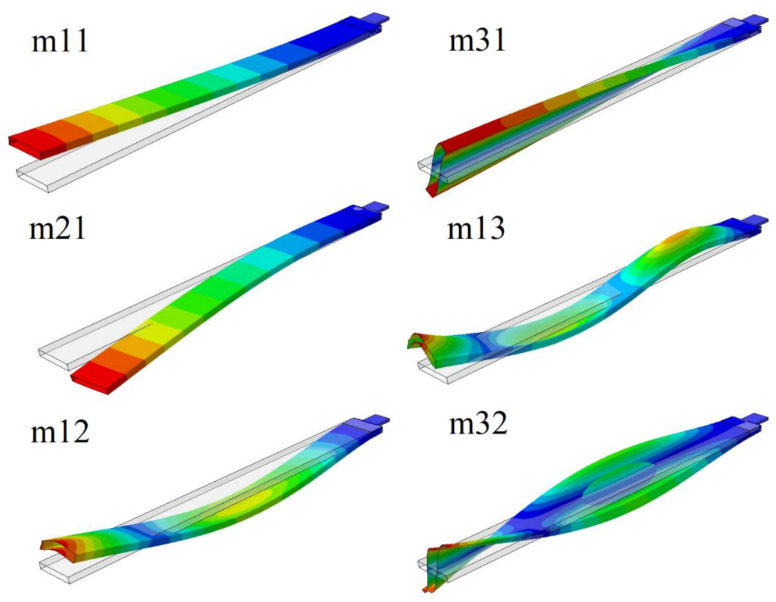
The global eigen-modes.

The eigen-frequencies and corresponding modes of the beam, with and without the hole, were determined using the numerical and experimental modal method. Figure 5 shows the selected global eigen-modes. These global modes were described by two numbers: the first one corresponded to the dominant effect: 1—bending in the flexible direction, 2—bending in the stiff direction, and 3—torsion; the second one was the number of modes in a certain direction. Table 1 lists the eigen-frequency values corresponding to the modes of the beam with hole (Case 2), and without the hole (Case 1), and their differences. The beam in Case2 had a hole with a radius of 8 mm, which was on the left line, at a distance of 350 mm from the fixed grip (denoted as beam 350L R8). A very good agreement was obtained for the first four modes (see modes denoted as m11, m21, m12, m31 in Table 1); however, for the next two modes (that is, the third flexible bending m31 mode and the second torsional m32 mode in Table 1), the differences were higher. For the first five modes, a similar effect of the hole on the change in eigen-frequency was noticed in numerical and experimental studies, whereas the sixth eigen-frequency decreased in the numerical studies but increased in the experimental one.

**Figure 6:**
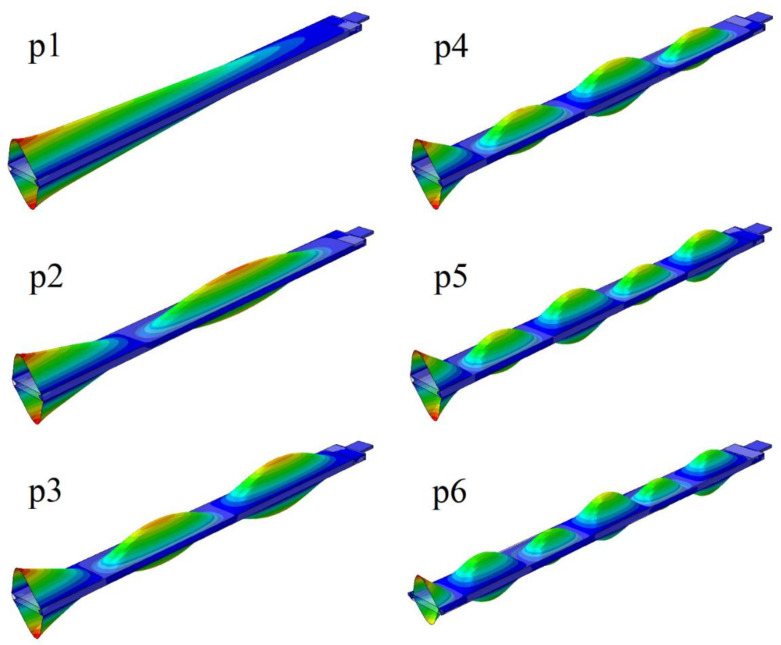
The local eigen-modes.

Furthermore, the local eigen-modes were considered, where deformations of the profile walls were dominant, without significant deformation of the beam axis. These deformations mainly occurred on the upper and lower beam walls because of their stiffness being significantly lesser than the side walls. Figure 6 shows the selected local modes. The number in the designation of these modes was equal to the number of visible nodal lines.

Table 2 presents the values of the eigen-frequencies in Hz corresponding to the analysed modes, both for the beam with (Case 2) and without the hole (Case 1). The twelve different locations of the 8 mm-radius hole were discussed. For the m11 mode, the natural frequency decreased when the prepared hole was closer to the grip and increased when it was closer to the free end of the beam. When the hole was close to the grip, the frequency values corresponding to the m21 mode significantly differed if its localization was on the central line (denoted as C), rather than the outside lines L or R (see Figure 1). These differences disappeared as the hole was placed farther from the grip. The frequency corresponding to the m12 mode decreased when the hole was closer to the ends of the beam (distances from the fixed grip: 150 or 750 mm) and increased when it was in the middle of the beam (distances from the fixed grip: 350 or 550 mm). The hole always caused the eigen-frequencies of both torsional modes (that is the m31 and m32 modes) to be lower relative to the frequencies of the beam without the hole. In case of the local modes, for the hole which was on the L or R lines, the values of the eigen-frequencies slightly increased or remained unchanged, whereas if the hole was on the central line, the frequencies for each local mode decreased significantly.

**Figure 7:**
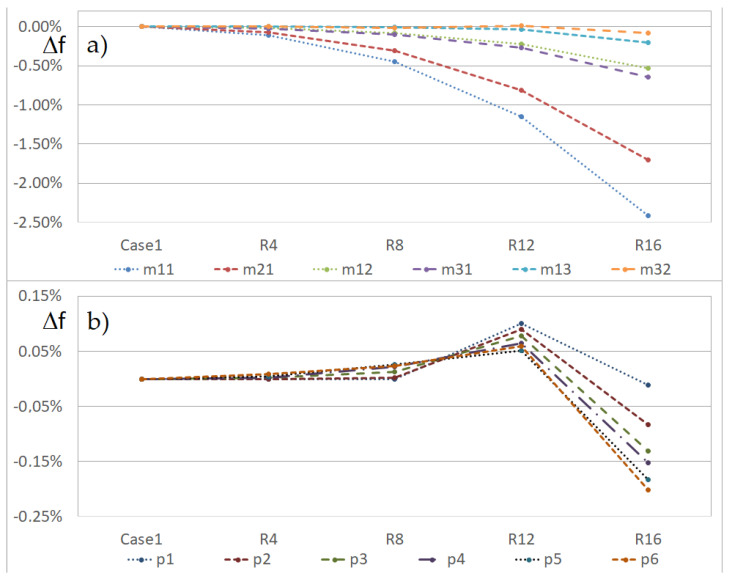
Percentage change in eigen-frequencies versus the hole radius (beam CUS 150L), (**a**) global, and (**b**) local eigen-modes.

Additionally, the influence of the hole radius on the change in the eigen-frequencies of the beam was examined. The radius of the hole was 4, 8, 12 or 16 mm. Table 3 and Figure 7a,b present the FEM results for the beam with the hole, which was on the L line at a distance of 150 mm from the grip. The hole radius was important for the first four eigen-frequencies. For some local modes, the small holes increased the eigen-frequencies, but the large one significantly decreased them.

**Figure 8:**
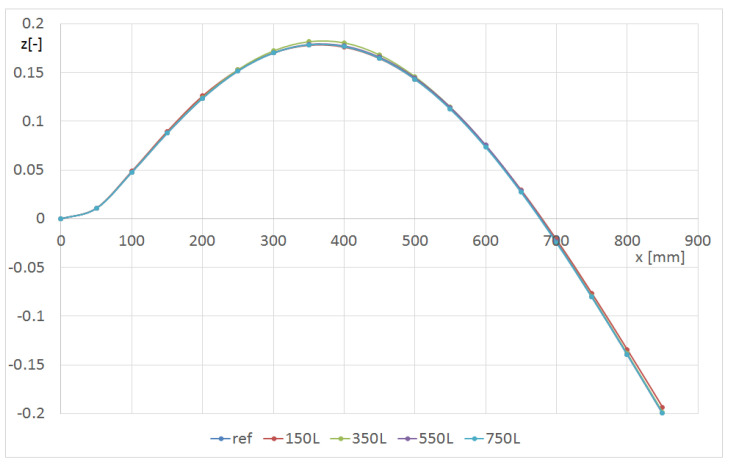
Shape of the L1 line with respect to the hole location for the m12 mode (beam CUS R8).

**Figure 9:**
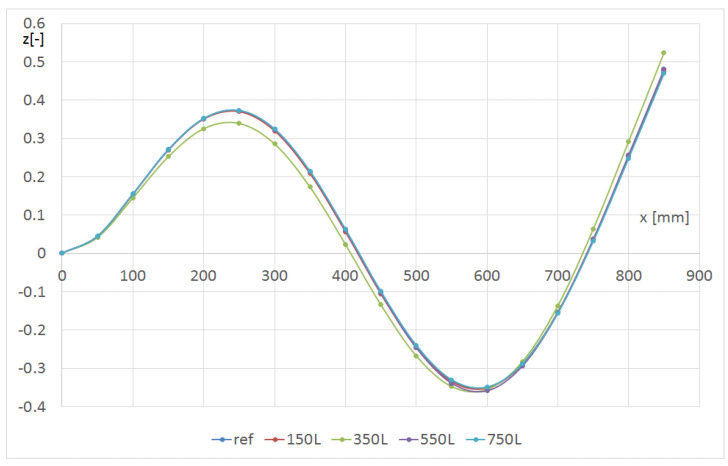
Shape of the L1 line with respect to the hole location for the m13 mode (beam CUS R8).

Next, a comparison of selected eigen-modes for the beam with and without the hole was discussed. Using FEM, the displacements of the points on the L1, L2 and L3 lines with respect to the hole radius and position were determined. Figure 8 and Figure 9 represent the influence of the hole position along the beam on the shape of the second and third bending modes in the flexible direction, respectively. The hole with a radius of 8 mm was on the L line (Figure 1). Only the line denoted as L1 was discussed, but for global modes, the shapes of lines L2 and L3 were similar. In both cases, the mode changed only if the hole was located 350 mm from the grip.

**Figure 10:**
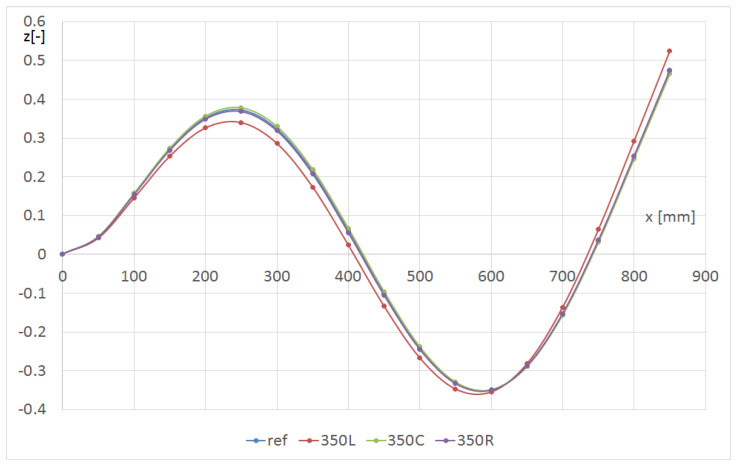
Shape of the L1 line with respect to the hole location in the transverse y-direction for the m13 mode (beam CUS R8).

Figure 10 shows the effect of the hole position in the transverse y direction on the shape of the L1 line for the m13 mode of the beam with the hole. The hole radius was always 8 mm (denoted as R8). The hole position along the beam was chosen to attain the greatest change in the beam deformation. The strongest influence of the hole was visible on the line closest to it.

**Figure 11:**
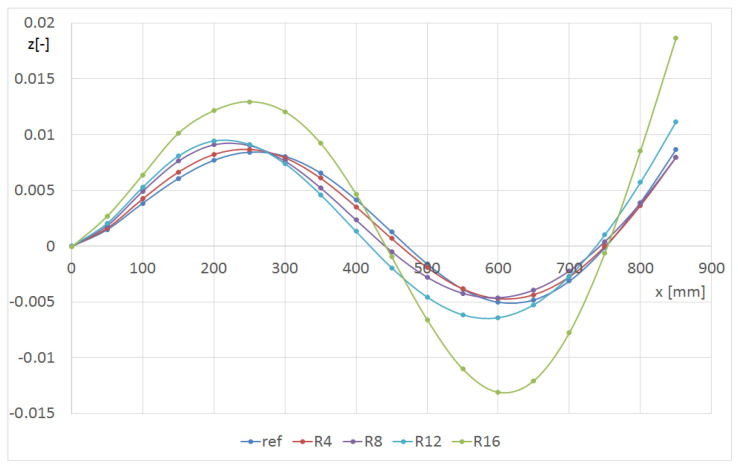
Shape of the L1 line with respect to the hole radius for the p2 mode (beam CUS 150L).

**Figure 12:**
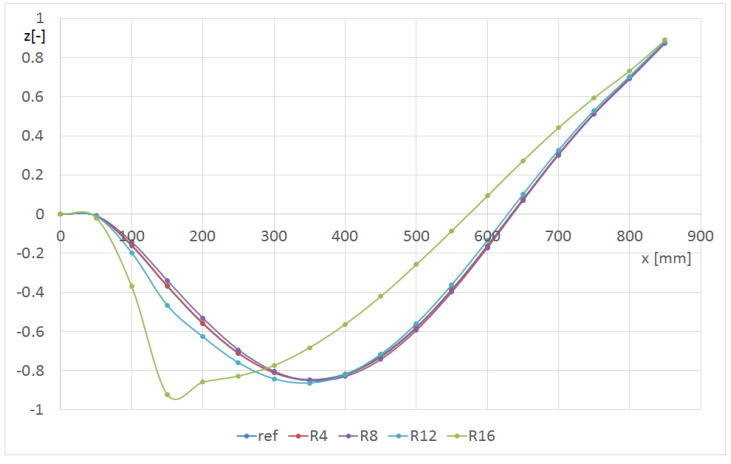
Shape of the L2 line versus the hole radius for the p2 mode (beam CUS 150L).

Figure 11 and Figure 12 show the effect of the hole radius on the p2 mode. The hole was on the L line at a distance of 150 mm from the grip. In Figure 11, the shape of the line L1 is presented, whereas in Figure 12, the line L2 is presented (central—Figure 1). The central L2 line (Figure 12) corresponded to the second flexure form, and the edge L1 line (Figure 11) corresponded to the third form with an amplitude that was fifty times lower. The maximum value of deformation at the end of the centreline was almost independent of the hole size. The deformations in the middle of the beam were abruptly changed in the damage’s area. For better observation, the hole radius should be at least 12 mm in diameter. When the L1 line was considered, the influence of the smaller hole was observed. The hole R16 caused a significant decrease in the local stiffness in the vicinity of the L2 line (the hole edge approached the line); therefore, the green curve visible in Figure 12 differs significantly from the others.

## 6. Conclusions

In this study, the effect of the radius and position of a hole made in a thin-walled cantilever beam on its dynamic response was tested. The selected numerical (FEM) results were validated using experimental modal analysis. The convergence of the model by selecting the size of the finite elements was investigated numerically, but mesh compaction did not reduce the errors between the experimental and numerical results. The differences in the results could be attributed to inaccuracies in the beam fabrication. Measurements at the edges showed slight variations in wall thickness.

The size and location of the hole affected the eigen-frequencies and the corresponding modes. The presented results revealed that the hole more intensively influenced the eigen-frequency corresponding to the low modes and the eigen-mode shape corresponding to the higher ones. To enable the assessment of the size and location of the hole, it was necessary to consider both global and local vibration modes. In this study, we showed that even a small hole in a beam could significantly change the shape of their local modes. The differences in the eigen-frequencies and corresponding modes of a beam with a hole increased progressively as with the radius of the hole.

## Figures and Tables

**Table 1:** Eigen-frequencies in Hz and their differences in % of the beam CUS 350L R8 with (Case 2) and without the hole (Case 1).

FEM				Experimental Results		
Modes	Case1	Case2	δ	Case1	Case2	δ
m11	33.601	33.540	−0.18%	35.996	35.821	−0.48%
m21	97.626	97.499	−0.13%	101.899	101.729	−0.17%
m12	194.230	194.120	−0.06%	190.606	190.275	−0.17%
m31	221.640	221.420	−0.10%	202.646	202.182	−0.23%
m13	359.680	359.810	0.04%	316.586	317.224	0.20%
m32	363.700	363.310	−0.11%	294.965	296.373	0.48%

**Table 2:** The hole localization effect on eigen-frequencies in Hz. The radius of the hole is 8 mm. FEM results.

		Distance from the Fixed Grip											
		150 mm			350 mm			550 mm			750 mm		
	Case1	Case2			Case2			Case2			Case2		
Modes	-	L	C	R	L	C	R	L	C	R	L	C	R
1 (m11)	33.60	33.45	33.43	33.41	33.54	33.54	33.53	33.61	33.61	33.60	33.65	33.65	33.65
2 (m21)	97.63	97.32	97.61	97.35	97.50	97.65	97.59	97.64	97.69	97.67	97.76	97.77	97.76
3 (m12)	194.23	194.07	194.19	194.16	194.12	193.98	193.77	193.97	193.82	193.57	194.26	194.23	194.21
4 (m31)	221.64	221.41	221.36	221.32	221.42	221.39	221.43	221.52	221.49	221.54	221.59	221.57	221.60
5 (m13)	359.68	359.64	359.69	359.84	359.81	359.67	359.40	359.02	359.47	359.73	359.62	359.45	359.42
6 (m32)	363.70	363.65	363.47	363.55	363.31	363.48	363.35	363.49	363.47	363.33	363.48	363.46	363.38
7 (p1)	455.82	455.82	455.43	455.82	455.84	455.37	455.84	455.89	455.28	455.90	455.97	455.15	456.00
8 (p2)	456.64	456.65	456.20	456.67	456.75	456.00	456.76	456.68	456.20	456.67	456.70	456.18	456.72
9 (p3)	458.42	458.48	457.90	458.50	458.44	458.00	458.43	458.58	457.79	458.62	458.42	458.03	458.42
10 (p4)	461.32	461.42	460.70	461.44	461.33	460.85	461.37	461.31	460.93	461.33	461.34	460.91	461.32
11 (p5)	465.77	465.89	465.08	465.90	465.86	465.10	465.83	465.91	465.14	465.90	465.87	465.22	465.84
12 (p6)	472.08	472.19	471.41	472.15	472.05	471.72	472.02	472.11	471.56	472.22	472.24	471.39	472.25

**Table 3:** Hole radius effect on eigen-frequencies in Hz. The hole is on left lines at a distance of 150 mm from the fixed grip. FEM results.

		Radius of the Hole			
Modes	Case1	R4	R8	R12	R16
1 (m11)	33.601	33.565	33.450	33.214	32.791
2 (m21)	97.626	97.554	97.322	96.832	95.968
3 (m12)	194.230	194.190	194.070	193.800	193.190
4 (m31)	221.640	221.580	221.410	221.040	220.210
5 (m13)	359.680	359.670	359.640	359.550	358.930
6 (m32)	363.700	363.690	363.650	363.730	363.390
7 (p1)	455.820	455.820	455.820	456.280	455.770
8 (p2)	456.640	456.640	456.650	457.050	456.260
9 (p3)	458.420	458.430	458.480	458.780	457.820
10 (p4)	461.320	461.330	461.420	461.620	460.620
11 (p5)	465.770	465.790	465.890	466.010	464.920
12 (p6)	472.080	472.120	472.190	472.360	471.130

## Data Availability

Data is contained within the article.

## References

[1] Jackson M., Shukla A. (2011). Performance of sandwich composites subjected to sequential impact and air blast loading. Compos. Part B Eng..

[2] Yang X., Swamidas A., Seshadri R. (2001). Crack identification in vibrating beams using the energy method. J. Sound Vib..

[3] Ashory M.-R., Ghasemi-Ghalebahman A., Kokabi M.-J. (2017). An efficient modal strain energy-based damage detection for laminated composite plates. Adv. Compos. Mater..

[4] Wronkowicz-Katunin A., Dragan K. (2018). Evaluation of Impact Damage in Composite Structures Using Ultrasonic Testing. Fatigue Aircr. Struct..

[5] Ding G., Song W., Gao X., Cao H. (2020). Damage Detection in Holed Carbon Fiber Composite Laminates Using Embedded Fiber Bragg Grating Sensors Based on Strain Information. Shock. Vib..

[6] Stawiarski A., Miarka S., Barski M., Romanowicz P. (2016). Fatigue Damage Detection in Composite Plate with a Circular Hole by Elastic Wave Propagation Method. Compos. Theory Pract..

[7] Gerges S., Sultan R., Younes M., Soaly E. (2012). Delamination Identification on Composite Material by Free Vibration Test. Int. J. Mech. Eng. Robot. Res..

[8] Kisa M. (2004). Free vibration analysis of a cantilever composite beam with multiple cracks. Compos. Sci. Technol..

[9] Lestari W., Hanagud S. (1999). Health Monitoring of Structures—Multiple Delamination Dynamics in Composite Beams. Structures, Structural Dynamics, and Materials and Co-Located Conferences, Proceedings of the 40th Structures, Structural Dynamics, and Materials Conference and Exhibit, St. Louis, MO, USA, 12–15 April 1999.

[10] Manoach E., Warminski J., Kloda L., Teter A. (2017). Numerical and experimental studies on vibration based methods for detection of damage in composite beams. Compos. Struct..

[11] Kessler S.S., Spearing S., Atalla M.J., Cesnik C.E., Soutis C. (2002). Damage detection in composite materials using frequency response methods. Compos. Part B Eng..

[12] Govindasamy M., Kamalakannan G., Kesavan C., Meenashisundaram G.K. (2020). Damage Detection in Glass/Epoxy Laminated Composite Plates Using Modal Curvature for Structural Health Monitoring Applications. J. Compos. Sci..

[13] Manson G., Worden K., Monnier T., Guy P., Pierce S., Culshaw B. (2009). Some Experimental Observations on the Detection of Composite Damage using Lamb Waves. Strain.

[14] Scalerandi M., Gliozzi A., Bruno C.L.E., Masera D., Bocca P. (2008). A scaling method to enhance detection of a nonlinear elastic response. Appl. Phys. Lett..

[15] Gürgen S., Sofuoğlu M.A. (2020). Vibration attenuation of sandwich structures filled with shear thickening fluids. Compos. Part B Eng..

[16] Gürgen S., Sofuoğlu M.A. (2020). Smart polymer integrated cork composites for enhanced vibration damping properties. Compos. Struct..

[17] Rehfield L.W., Atilgan A.R., Hodges D.H. (1990). Nonclassical Behavior of Thin-Walled Composite Beams with Closed Cross Sections. J. Am. Helicopter Soc..

[18] Latalski J., Bochenski M., Warminski J. (2015). Control of Bending-Bending Coupled Vibrations of a Rotating Thin-Walled Composite Beam. Arch. Acoust..

[19] Teter A., Gawryluk J. (2016). Experimental modal analysis of a rotor with active composite blades. Compos. Struct..

[20] Abaqus 6.14 HTML Documentation. http://130.149.89.49:2080/v6.14/.

[21] Teter A., Gawryluk J., Bocheński M. (2018). Experimental and numerical studies of a cracked thin-walled box-beams. Compos. Struct..

[22] Gawryluk J., Bocheński M., Teter A. (2017). Modal Analysis of Laminated “CAS” and “CUS” Box-Beams. Arch. Mech. Eng..

[23] Barbero E.J. (2015). Finite Element Analysis of Composite Materials Using Abaqus^TM^.

